# Boosting autophagy in anti-tumor proteasome inhibition-mediated cardiotoxicity

**DOI:** 10.18632/aging.204724

**Published:** 2023-05-08

**Authors:** Eleni-Dimitra Papanagnou, Sentiljana Gumeni, Ioannis P. Trougakos

**Affiliations:** 1 Department of Cell Biology and Biophysics, Faculty of Biology, National and Kapodistrian University of Athens, Athens 15784, Greece

**Keywords:** autophagy, cardiotoxicity, proteasome dysfunction, proteasome inhibitors, proteotoxicity

Proteome stability (also referred to as proteostasis) is critical for proper cellular functionality and consequently organismal health and it is ensured by an extensive compartment-specific network of machineries known as the proteostasis network (PN) [1]. Key components of the PN are the two main proteolytic machineries, namely the autophagy lysosome- (ALP) and the ubiquitin proteasome- (UPP) pathways. ALP is an essential, conserved cellular process that (among others) degrades damaged organelles and protein aggregates being comprised by microautophagy, chaperone-mediated autophagy and macroautophagy [1]. On the other hand, UPP is central to protein synthesis quality control; it is also responsible for ∼80% of the total protein turnover as it degrades short-lived and also misfolded or damaged peptides. Considering the large number of proteins being degraded by the proteasome, it is not surprising that UPP modulates essential cellular processes including cell cycle, gene expression, responses to growth factors, cellular stress and metabolism.

Declined proteasome functionality is a hallmark of aging and a major risk factor for the development of many age-related diseases such as neurodegenerative disorders and cardiomyopathies [2]. On the other hand, high levels of proteasome activity can be crucial for cancer cells survival, likely due to tumor-related excessive proteome instability and oxidative damage [3]. Therefore, proteasome inhibition provides a promising anti-tumor therapy [4]. Although, proteasome inhibitors (PIs) like Bortezomib and Carfilzomib, have revolutionized the treatment of hematologic malignancies (e.g., multiple myeloma), a major limitation in the clinic is the emergence of severe side effects such as cardiotoxicity; specifically, the heart-specific adverse effects of PIs include arrhythmia, hypertension, ischemic heart disease, cardiomyopathy, pulmonary hypertension, heart failure, thromboembolic events, and rarely sudden cardiac death [4]. Cardiomyocytes are terminally differentiated post-mitotic cells, with limited regenerative capacity and constant exposure to various types of stress including metabolic, proteotoxic, oxidative and mechanical stress. Therefore, cardiomyocytes are highly dependent on proper PN functionality and are thus increasingly sensitive to PIs administration [4]. Despite the growing evidence regarding the PIs-related cardiotoxicity, the underlying mechanistic details of PIs-mediated cardiovascular complications remain largely elusive.

**Figure 1:**
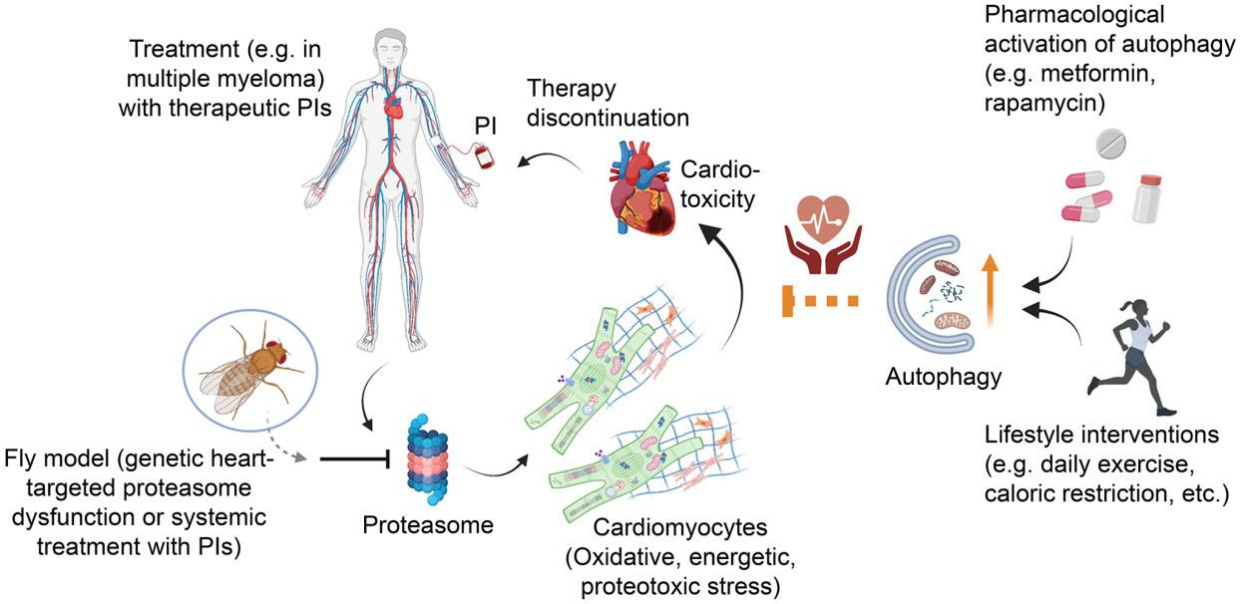
**Autophagy enhancement attenuates proteasome dysfunction–mediated cardiotoxicity.*** Drosophila* bears a heart-like structure and a circulatory system with developmental and functional homologies to the vertebrates' heart and circulation. Genetic heart-targeted proteasome dysfunction or exposure of flies to systemic anti-tumor therapeutic PIs triggers increased oxidative, proteotoxic and energetic stress in cardiac tissues resulting in (among others) mitochondrial perturbation, low energy levels and eventually severe cardiotoxicity. Although less studied, these consecutive events that trigger systemic toxicity and cardiac complications can be also seen in tumor patients treated with PIs, leading to therapy discontinuation and a negative survival outcome. As found in the fly model [6] proteasome dysfunction-mediated cardiotoxicity can be partially rescued by pharmacological co-treatment with autophagy activators such as metformin/rapamycin or by caloric restriction; in humans additional lifestyle adaptations (e.g. daily exercise) can be also beneficial.

By exploiting the *Drosophila* experimental *in vivo* model we previously reported that flies exposed to therapeutic PIs (i.e., Carfilzomib or Bortezomib) display similar adverse effects with multiple myeloma patients treated with PIs, including both neurotoxicity and cardiac dysfunction [5]. Recently, we genetically knocked down proteasome functionality only in the fly heart and found a generalized collapse of both proteome and mitochondria functionality in the heart tissue likely due to the high levels of proteotoxic, oxidative and metabolic stress [6]; these molecular defects led to developmental abnormalities and growth retardation while in the adult they disrupted cardiac activity, eventually causing systemic toxicity and reduced longevity. Similarly, flies treated with systemically administered PIs exerted increased proteome instability, mitochondrial dysfunction, cardiotoxicity and acceleration of aging-related phenotypes [6]. Interestingly, the heart-specific proteasome knockdown deleterious effects were attenuated following autophagy activation via genetic (e.g., heart-targeted Atg8a/LC3B overexpression), dietary (e.g., low protein intake) or pharmacological (e.g., rapamycin or metformin -an EMA/FDA approved drug- administration) interventions, hence highlighting the importance of UPP and ALP functional interplay for proteome damage dilution (Figure 1). The alleviation of the proteasome dysfunction-induced cardiotoxic and pro-aging phenotypes following autophagy activation were linked to the partial restoration of both cellular proteostasis and mitostasis, leading thus to energetics rebalance and reduction of the proteotoxic damage (see also, [7]). In support, a recent study in the mouse model showed that metformin, improved several adverse effects caused by PIs probably by restoring AMPKα phosphorylation and overall, the autophagic flux [8].

Taken together, our findings provide mechanistic explanations for the heart-related adverse effects of therapeutic PIs, suggesting that proteasome dysfunction in the heart results in cardiotoxicity and systemic complications due to proteome instability, mitochondria disruption, redox imbalance and metabolic deregulation. Moreover, our therapeutically relevant finding that co-administration of autophagy inducers (e.g., metformin) exert beneficial effects on heart functionality, provide preclinical insights for alleviating PIs-induced cardiac adverse effects and thus preventing anti-tumor therapy discontinuation.
